# Application of an indoor air pollution metamodel to a spatially-distributed housing stock

**DOI:** 10.1016/j.scitotenv.2019.02.341

**Published:** 2019-06-01

**Authors:** Jonathon Taylor, Clive Shrubsole, Phil Symonds, Ian Mackenzie, Mike Davies

**Affiliations:** a UCL Institute for Environmental Design and Engineering, Central House, 14 Upper Woburn Plc, London WC1H 0NN, UK; b University of Edinburgh School of GeoSciences, Crew Building, The King's Buildings, Alexander Crum Brown Road, Edinburgh EH9 3FF, UK

**Keywords:** Building physics, I/O ratios, Air pollution, PM_2.5_, NO_2_

## Abstract

Estimates of population air pollution exposure typically rely on the outdoor component only, and rarely account for populations spending the majority of their time indoors. Housing is an important modifier of air pollution exposure due to outdoor pollution infiltrating indoors, and the removal of indoor-sourced pollution through active or passive ventilation. Here, we describe the application of an indoor air pollution modelling tool to a spatially distributed housing stock model for England and Wales, developed from Energy Performance Certificate (EPC) data and containing information for approximately 11.5 million dwellings. First, we estimate indoor/outdoor (I/O) ratios and total indoor concentrations of outdoor air pollution for PM_2.5_ and NO_2_ for all EPC dwellings in London. The potential to estimate concentration from both indoor and outdoor sources is then demonstrated by modelling indoor background CO levels for England and Wales pre- and post-energy efficient adaptation, including heating, cooking, and smoking as internal sources. In London, we predict a median I/O ratio of 0.60 (99% CIs; 0.53–0.73) for outdoor PM_2.5_ and 0.41 (99%CIs; 0.34–0.59) for outdoor NO_2_; Pearson correlation analysis indicates a greater spatial modification of PM_2.5_ exposure by housing (ρ = 0.81) than NO_2_ (ρ = 0.88). For the demonstrative CO model, concentrations ranged from 0.4–9.9 ppm (99%CIs)(median = 3.0 ppm) in kitchens and 0.3–25.6 ppm (median = 6.4 ppm) in living rooms. Clusters of elevated indoor concentration are found in urban areas due to higher outdoor concentrations and smaller dwellings with reduced ventilation potential, with an estimated 17.6% increase in the number of living rooms and 63% increase in the number of kitchens exceeding recommended exposure levels following retrofit without additional ventilation. The model has the potential to rapidly calculate indoor pollution exposure across large housing stocks and estimate changes to exposure under different pollution or housing policy scenarios.

## Highlights

• A housing stock model representing 11.5 M English and Welsh dwellings is described.
• An IAQ metamodel is applied to dwellings in London (PM_2.5_ & NO_2_) and nationally (CO).
• Exposure to outdoor NO_2_ & PM_2.5_ and outdoor and indoor-sourced CO is estimated.
• Housing has a greater modification of outdoor PM_2.5_ levels than for NO_2_.
• Energy retrofits without added ventilation may increase CO exposures by 18–63%.

## Graphical abstract

**Figure:**
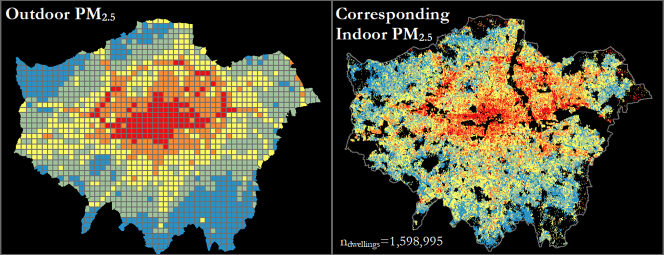

## 1 Introduction

Air pollution exposure is one of the largest contributors to premature mortality in the UK, with around 40,000 deaths brought forward attributable to exposure to particulate air pollution and NO_2_ annually (RCP, 2016). Background levels of air pollution also have implications for morbidity. Air pollution exposure has been associated with a number of physiological diseases (RCP, 2016), while exposure to even low concentrations of indoor pollutants, such as carbon monoxide (CO), has been linked with neurological symptoms in building occupants (Croxford et al., 2008).

Housing and occupant behaviour are important modifiers of air pollution exposure, with building characteristics such as geometry and design, permeability, and ventilation components impacting on the infiltration of outdoor pollution indoors and the removal of internally-generated pollution (Taylor et al., 2014; Shrubsole et al., 2012; Taylor et al., 2016; Fabian et al., 2012; Dimitroulopoulou et al., 2006). Given that the UK population spends around 90% of their time indoors (ONS, 2005), buildings – particularly the housing which is predominantly mixed-mode – are an important microenvironment for pollution exposure (Smith et al., 2016). Due to the significant health-burden of air pollution in the UK, there is a need to understand population indoor exposures, taking into account spatial variations in both outdoor pollution levels and the modifying effects of the housing stock. In addition, there is a growing need to quantify changes in indoor exposure following policy-driven changes to the housing stock - for example dwelling energy efficiency improvements - and outdoor pollution levels.

Indoor air pollution is often estimated using deterministic models, whereby exposure is modelled as a function of building geometry, ventilation characteristics, outdoor concentrations, indoor emission strengths and schedules, and the physical properties of the pollutants [e.g. (Shrubsole et al., 2012; Fabian et al., 2012; Emmerich et al., 2005; Hamilton et al., 2015; Milner et al., 2011)]. To evaluate indoor exposure at the population-level, models must be run for housing variants representative of the stock. A number of studies have sought to estimate population-level indoor pollution exposure at the regional or national level in the UK. Taylor et al. (2014) estimated indoor concentrations of PM_2.5_^2^ for London by combining modelled outdoor levels with building physics-derived estimates of indoor/outdoor (I/O) ratios in a geographically-referenced housing stock model. While the modelled dwelling archetypes were representative of 76% of the London housing stock, variations in dwelling size or building fabric properties within the archetypes were not considered. A similar approach was taken in a subsequent work, which estimated indoor pollution exposure across Great Britain, modelling I/O ratios and concentrations of air pollutants from indoor sources (Taylor et al., 2016). Housing information was obtained for approximately 1 million homes at postcode-level via the Homes Energy Efficiency Database (HEED), and building physics used to model unique combinations of dwelling geometry and building fabrics. Due to the very large number of simulations required, this study did not vary dwelling size, nor did it investigate any changes to exposure following energy efficient adaptations to the housing stock. Other studies have sought to estimate population-level exposure indoors by estimating concentrations across a representative but non-geographically referenced housing stock. Hamilton et al. (2015) used building simulation to estimate indoor exposure to radon, PM_2.5_, environmental tobacco smoke, cold, and damp for England at the population-level using a representative housing stock model. Changes to exposure following a number of energy efficiency interventions were estimated, which were, in turn, converted to health outcomes. However, this study used a limited number of dwelling archetypes, and did not account for the spatial variation in housing types and their local outdoor pollution concentrations. Internationally, a number of studies have used building modelling approaches to estimate the spatial distribution of indoor air pollution exposure. Building infiltration rates have been estimated for dwellings in different US regions (Persily et al., 2010), which have then been used to estimate indoor exposure to outdoor PM_10_ in these regions (Chen et al., 2012). Sarnat et al. (2013) included spatially-varying estimates of building air exchange rates and outdoor NOx, CO, and PM_2.5_ concentrations for Atlanta, comparing estimated indoor exposures to hospital visits for asthma and wheeze.

Owing to the wide availability of large air pollution datasets, machine learning techniques are increasingly being used to estimate air pollution exposures (Bellinger et al., 2017). While the vast majority of such studies examine outdoor air pollution, machine learning has been applied to estimate indoor exposures to outdoor and ground-sourced pollution given monitored indoor NO_2_ and PM_2.5_ (Challoner et al., 2015), radon (Pegoretti and Verdi, 2009) and modelled PM_2.5_ (Dias and Tchepel, 2014; Symonds et al., 2016). Symonds et al. (2016) developed a neural network modelling framework for both indoor temperatures and PM_2.5_ from outdoor sources using the outputs of building physics models. In both (Symonds et al., 2016) and other building simulation studies (Van Gelder et al., 2014), neural networks performed better than other metamodeling techniques such as Support Vector Machines (SVMs). However, other machine learning techniques may perform better in other cases. This neural network framework provides opportunities to overcome the computational limitations of the above building physics-based studies (Taylor et al., 2014; Taylor et al., 2016; Hamilton et al., 2015) – where an individual simulation of a dwelling may take several minutes to an hour, depending on the building complexity and computational power. This approach facilitates the rapid calculation of indoor air pollution exposure at the housing stock level given detailed sets of housing characteristics under a range of different housing scenarios.

In this study, we describe the application of this metamodeling framework across a large geographically-referenced housing stock model to predict indoor air pollution levels. The objectives are to:

1) Develop an underlying geographically-referenced housing stock model using the recently-released Energy Performance Certificate (EPC) data (DCLG, 2017) as input to the metamodel.
2) To apply the metamodeling framework described previously (Symonds et al., 2016), and further adapted for this study, to predict the I/O ratios of outdoor PM_2.5_ and NO_2_ for all EPC dwellings in London; and to overlay these I/O ratios with modelled ambient outdoor air pollution concentrations to estimate total levels of indoor exposure to background outdoor air pollution.
3) To demonstrate the potential of the model to estimate total levels of pollution concentration from both indoor and outdoor sources, for individual buildings in England and Wales. Here, background levels of CO are modelled along with internal sources from heating, cooking, and smoking both prior to and following building retrofit. The model application here is demonstrative, as there is significant uncertainty in indoor emission rates. CO was selected as the modelled pollutant as its deposition rate is negligible, removing this additional level of uncertainty.

The approach described here offers a number of improvements over the previous studies that sought to model regional or national indoor air pollution in the UK mentioned above, including better coverage and detail of housing data – including potential indoor pollution sources - along with the ability to rapidly examine a range of different housing and pollutant emission scenarios.

## 2 Methods

**Fig. 1:**
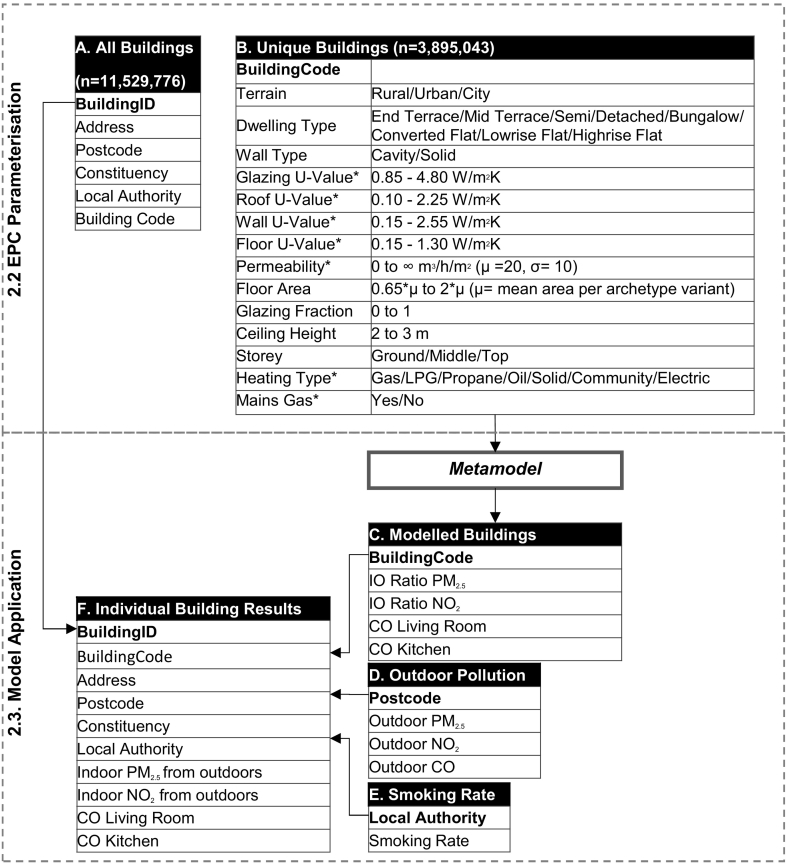
Individual dwellings in the EPC database are parameterised, and split into a main dataset with locational information (table A) and a dataset with unique combinations of metamodel input variables selected (table B) linked by a BuildingCode. These unique combinations are modelled using the metamodel. The model outputs (C) are then joined back to table A, while outdoor pollution concentrations (table D) are joined by postcode and smoking rates (table E) joined by local authority.

The modelling workflow and input data for the metamodel can be seen in Fig. 1, and are described in the corresponding sections below.

### 2.1 Metamodel

The neural network metamodel is an updated version of the modelling framework described previously by Symonds et al. (2016); we refer the reader to that paper for full details of the development and performance of the applied model. Briefly, the framework consists of metamodels for eight different building archetypes, representative of the English housing stock (Appendix 1). These archetypes were derived for previous stock modelling, and have built forms and internal layouts representative of the average English dwelling (Taylor et al., 2016).

The basis of each metamodel is a large number of EnergyPlus (US-DOE, 2013) simulations of indoor air pollution for the archetypes using the Generic Contaminant Model. Building parameters for the EnergyPlus models were randomly generated from pre-defined distributions using a Latin Hypercube design (Fig. 1). The choice of distribution, along with the mean and range of the input parameters were informed by nationally representative housing stock surveys such as the English Housing Survey (EHS) (DCLG, 2011). The resulting EnergyPlus models dynamically simulate air change rates based on hourly wind conditions, terrain-related wind exposure, and building characteristics including fabric permeability (infiltration) and thermal performance (buoyancy). Internal emissions and occupancy were modelled using fixed schedules, described in Section 2.3.2. The models were run with a time-step of 10 min (NO_2_ and PM_2.5_) or 5 min (CO) on a High Performance Computer (HPC) system at UCL, outputting hourly average indoor air pollution levels for each room in the dwelling for a year. A neural network modelling framework was then developed using PyBrain (Schaul et al., 2010), relating the randomly-sampled EnergyPlus input parameters to indoor air pollution metrics calculated from the simulation results. For externally-sourced NO_2_ and PM_2.5_, the metamodeling framework estimates the annual average I/O ratio based on the assumed time-activity profile of the occupants (two individuals, home all day) within the dwelling. Occupants are assumed to spend waking hours in the living room (7 am–10 pm), and night time hours (10 pm–7 am) in the primary bedroom. For CO, it estimates the annual maximum 8-hour mean concentration inside dwellings for the living room and kitchen, which enables comparison with the WHO-recommended exposure threshold of 8.1 ppm over this period (WHO, 2010).

Advances to the metamodel used in this paper include the ability to vary floor area, ceiling height, and glazing ratio of dwellings, and the capacity to vary pollutant indoor emissions rates using a power law distribution informed by CO emissions in the PANDORA dataset (Abadie and Blondeau, 2011). The deposition velocity of the generic indoor contaminant has been added as an additional metamodel variant, allowing predictions for multiple pollutants (NO_2_ and PM_2.5_) to be made, one at a time. The original model execution has also adapted and improved to allow additional dwelling input data on mains gas connectivity and heating type to flag potential indoor sources of air pollution and their emission rates. Internal layouts are held constant.

### 2.2 Building stock data

Domestic building stock data for England and Wales was obtained from the EPC database (DCLG, 2017). The EPC database contains information on dwelling geographical location – including postcode and constituency – and housing characteristics related to energy efficiency, which is gathered when a dwelling is sold, rented, or undergoes an energy-efficiency retrofit. To provide the underlying housing stock data required as input to the metamodels, the EPC database was parameterised. The process of parameterisation is described in detail in Appendix 2. Briefly, the raw EPC data was converted into metamodel inputs (Fig. 1) through a process of data cleaning, and dwelling energy efficiency and permeability estimated using the UK Governments Standard Assessment Procedure for energy in buildings (SAP) (BRE, 2009) using a SAS (SAS Institute, 2017) script. We acknowledge significant uncertainty in the SAP methods. Parameterisation followed methods outlined in previous work that has converted housing survey data for energy performance calculations (Hughes et al., 2012). In addition, the EPC data was parameterised a second time to represent the complete retrofit of the housing stock to increase energy efficiency, reflecting changes to building fabric thermal efficiency and airtightening. Fabric U-values were reduced to the minimum possible for dwelling age and fabric type according to SAP (Taylor et al., 2018). Reductions in airtightness were estimated first as changes to the dwelling air change rate (ach) following floor sealing and draught-stripping (using the reductions specified in the SAP model), or cavity wall, solid wall, or loft insulation (using estimated reductions from Hong et al. (2004)). The dwelling permeability was then re-estimated from the ach as in SAP.

The parameterised EPC data was then filtered to remove multiple instances of the same dwelling by selecting buildings by the building reference number with the most recent inspection date. Dwellings with missing data were removed. This resulted in 11,529,776 unique records (Fig. 1A) (summarised in Table 1). To reduce the metamodel processing time, 3,895,043 unique instances of dwellings were selected (Fig. 1), linked to the original database using a BuildingCode.

**Table 1:** Summary for EPC dwelling characteristics.

Parameter	Value	Percent
Dwelling type	End Terrace	9.2
	Mid Terrace	19.3
	Semi	23.3
	Detached	14.0
	Bungalow	9.4
	Converted Flats	23.5
	Low rise Flats	1.2
	High rise Flats	0.1
Wall type	Cavity	66.2
	Solid	33.8
Terrain	City	35.9
	Urban	60.8
	Rural	3.3
Main fuel	Gas	80.9
	Kerosene	0.7
	LPG/Propane	3.8
	Solid	0.9
	Electric/Community	13.7

In order to evaluate the representativeness of the parameterised EPC data, a random stratified selection of 1,000,000 EPC certificates was sampled from the dataset and the converted results compared by region against the EHS, which is representative of the English housing stock. Comparisons of the EPC dataset and the representative EHS showed good agreement (Appendix 2), with a slight skew of the EPC towards energy efficient dwellings. This provided confidence that the EPC data is representative of the English housing stock as a whole.

**Fig. 2:**
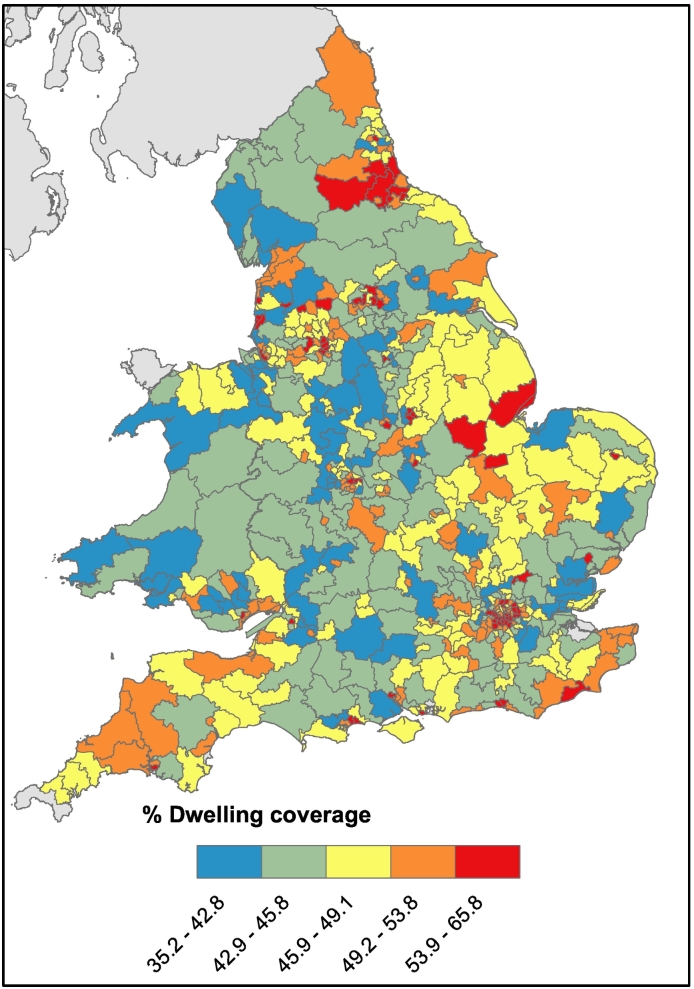
Coverage of EPC dwellings across England and Wales by constituency.

The coverage of the EPC data was evaluated by summing the unique building reference numbers by postcode and constituency. At the postcode level, there was buildings data in 1,173,614 postcodes (or 77.4% of English and Welsh postcodes), with a median of 7 and mode of 1 in each postcode. The constituency sum was used to estimate a percent coverage by comparing it with the number of dwellings in each constituency according to the 2011 Census (ONS, 2011), and mapped using ArcGIS (ESRI, 2017). The parameterised data covered an estimated 47% of the 24.4 million dwellings across England and Wales. The coverage across England and Wales can be seen in Fig. 2, while the variation in housing characteristics is illustrated in Appendix 2. EPC dwelling coverage in constituencies range from 0 to 66% in Nottingham South (median 46.3%).

### 2.3 Model application

The metamodel was applied in London using the parameterised EPC dataset for PM_2.5_ and NO_2_ from outdoor sources, and in England and Wales for CO from both indoor and outdoor sources. The underlying EnergyPlus models were run with static occupant behaviour assumptions, with summertime window-opening modelled to occur when modelled daytime indoor temperatures exceed 23 °C and night time temperatures exceed 21 °C; indoor heating was modelled using a thermostat setting of 22 °C from September–May. These are informed by comfort standards detailed in the Chartered Institute of Building Service Engineers (CIBSE) Guide A (CIBSE, 2015).

#### 2.3.1 Estimates of indoor concentration of outdoor PM_2.5_ and NO_2_ in London

Dwellings located in London were selected from the parameterised EPC dataset (1,598,995 dwellings, or approximately 44% of the total current London stock), and the metamodel used to estimate an annual average I/O ratio for PM_2.5_ and NO_2_. Both PM_2.5_ and NO_2_ were modelled with deposition velocities to account for variations in building geometry. The English-average ratio of the internal surface area (floor, ceiling, and total wall area) to internal volume (floor area × ceiling height) was estimated to be 2.3 m^−1^ using data from the EHS. Deposition rates for PM_2.5_ (0.19 h^−1^) (Long et al., 2001) and NO_2_ (0.87 h^−1^) (Emmerich and Persily, 1996) were adjusted by this value to estimate their deposition velocities, 2.26 × 10^−5^ m s^−1^ and 1.04 × 10^−4^ m s^−1^, respectively. PM_2.5_ was modelled with a penetration factor (defined as the fraction of pollutants that infiltrate through the building envelope) of 0.8 during the heating season, and 1 during the summer, while NO_2_ was modelled with a fixed penetration factor of 1 (Fabian et al., 2012). The model does not currently allow for seasonal or daily changes in outdoor air pollution or dwelling I/O ratio. We acknowledge significant uncertainty in the penetration rates and deposition velocities of the pollutants.

Background annual average PM_2.5_ and NO_2_ levels for 2015 were obtained from the UK Department for Environment, Food and Rural Affairs (DEFRA) website in a 1 km × 1 km grid for London (DEFRA, 2015). These outdoor values were spatially joined to London postcode boundaries in ArcGIS, and matched to the modelled EPC dwellings; the outdoor levels were then multiplied by the modelled I/O ratio of individual dwellings to estimate total indoor concentration of outdoor air pollution. The Pearson correlation coefficient between indoor and outdoor pollution was then calculated to estimate the modifying effect that dwellings have for both PM_2.5_ and NO_2_. Multicollinearity in outdoor pollution caused by gridded DEFRA data spanning multiple postcodes was addressed by merging postcodes that shared an underlying grid cell.

#### 2.3.2 National estimates of indoor CO levels

**Fig. 3:**
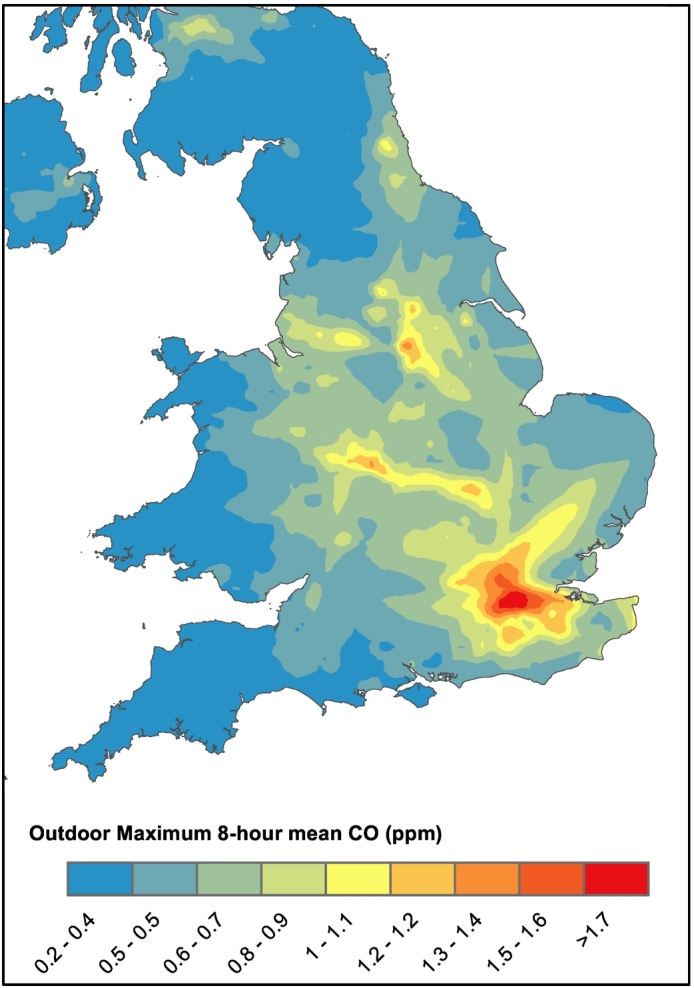
Outdoor annual maximum 8-hour mean CO concentration (ppm) modelled by Vieno et al. (2016).

To demonstrate the ability to the model to estimate concentration from outdoor and indoor sources, CO concentration was estimated for outdoor sources, and indoor cooking, smoking, and heating systems. The negligible deposition rate of CO with a penetration factor of 1 means that the I/O ratio without indoor sources approximates to 1. Therefore, the metamodel was used to model the background indoor concentration from indoor sources, while local 8-hour annual maximum outdoor concentrations were extracted from modelled values (Fig. 3, see Vieno et al. (2016) and references therein for model description). These were summed to estimate the maximum theoretical 8-hour concentration from both indoor and outdoor sources in dwellings.

We focus on internal emissions under normal operation (indoor levels from 0–30 ppm) rather than defective appliances that may cause short-term health problems (levels above 100 ppm). We acknowledge a great deal of uncertainty in emission rates and occupant activities which may lead to indoor CO generation; therefore, the model is intended to be demonstrative of the relative effects of housing on indoor concentration rather than to produce absolute estimates. Indoor CO emission rates for the different activities can be seen in Table 2. We assumed a working extract fan in the kitchen during cooking, and that no supplemental ventilation is provided during smoking. For heating, we assumed 90% of CO is vented outside, an estimate informed by comparing initial source-specific model outputs with values from the literature (Humfrey et al., 1996). The schedule of pollutant-generating activities has been taken from previous studies into indoor air pollution in English dwellings (Shrubsole et al., 2012; Taylor et al., 2016; Hamilton et al., 2015) (Table 2). Emissions from indoor heat sources (living room) were assumed to occur during heating hours when the indoor temperatures drop below the thermostat setpoint of 22 °C, which represents the lower range of the recommended thermal comfort criteria for UK living rooms (CIBSE, 2015). Emission rates were assumed to be constant while activities were occurring.

**Table 2:** CO emission activity schedules, additional provided ventilation, and emission rates for different indoor sources.

Activity	Times	Location of CO source	Ventilation	Fuel/heating type	Estimated emissions rate (mg/min)	Reference
Heating	06:00–08:00, 16:00–24:00, Sept–May	Living room	Assumed 90% vented	Gas (mains)	15.9	(Cáceres et al., 1983; Girman et al., 1982)
				Bulk LPG or Bottled gas – propane	8.7	(Cáceres et al., 1983)
				Heating oil	1.7	(Cáceres et al., 1983)
				House coal, wood or solids	2889	(Tissari et al., 2008)
				Community or electric	–	–
Cooking	07:40–08:00, 19:00–19:30	Kitchen	Extract fan (0.06 m^3^/s)	Gas	29	(Dimitroulopoulou et al., 2006)
				Electric	–	–
Smoking	5 min per hour, 08:00–22:00	Living room	None	Smoker	7.2	(Dimitroulopoulou et al., 2006)
				Non-smoker	–	–

The primary heating system of EPC dwellings was used to flag the housing heating system and modify the CO emission rate from heating accordingly. Similarly, if the EPC dwelling was not connected to mains gas, it was assumed that an electric stove was used for cooking. The metamodel framework was run for all unique EPC dwellings assuming both smoking and non-smoking households. The results were then joined back to the main parameterised EPC database, with results with and without indoor smoking weighted according to the estimated number of smoking households in each local authority taken from the UK Office for National Statistics (ONS, 2017).

## 3 Results

### 3.1 Indoor concentrations of outdoor pollution in London

**Fig. 4:**
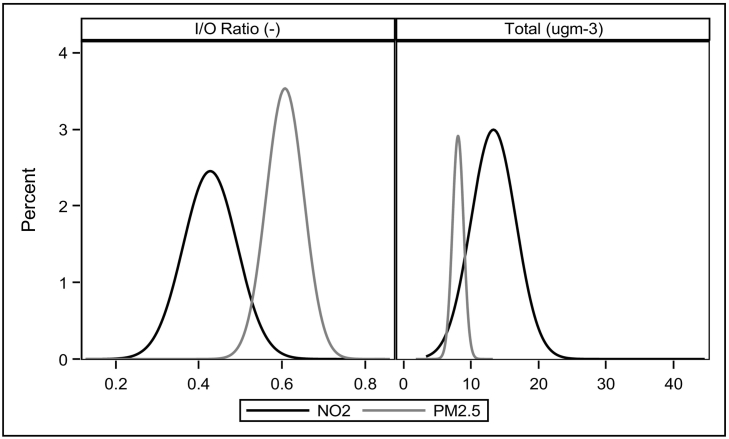
Distribution of individual-dwelling I/O ratios and total concentration of indoor pollution from outdoor sources for NO_2_ and PM_2.5_.

Outdoor levels of NO_2_ vary from 9.9–53.7 μg m^−3^ (median exposure 25.7 μg m^−3^), while outdoor PM_2.5_ varies from 8.6–14.6 μg m^−3^ (median exposure 11.1 μg m^−3^). The modelled distributions of I/O ratios and absolute concentrations can be seen in Fig. 4. Individual dwelling modelled I/O ratios for NO_2_ had a median of 0.41 (99% CIs; 0.34–0.59), while I/O ratios for PM_2.5_ had a median of 0.60 (99% CIs; 0.53–0.73). These lead to ranges of indoor exposure for NO_2_ of 7.3–23.3 μg m^−3^ (99% CIs; median 12.9 μg m^−3^), and for PM_2.5_ of 6.4–10.2 μg m^−3^ (99% CIs; median 8.0 μg m^−3^).

**Fig. 5:**
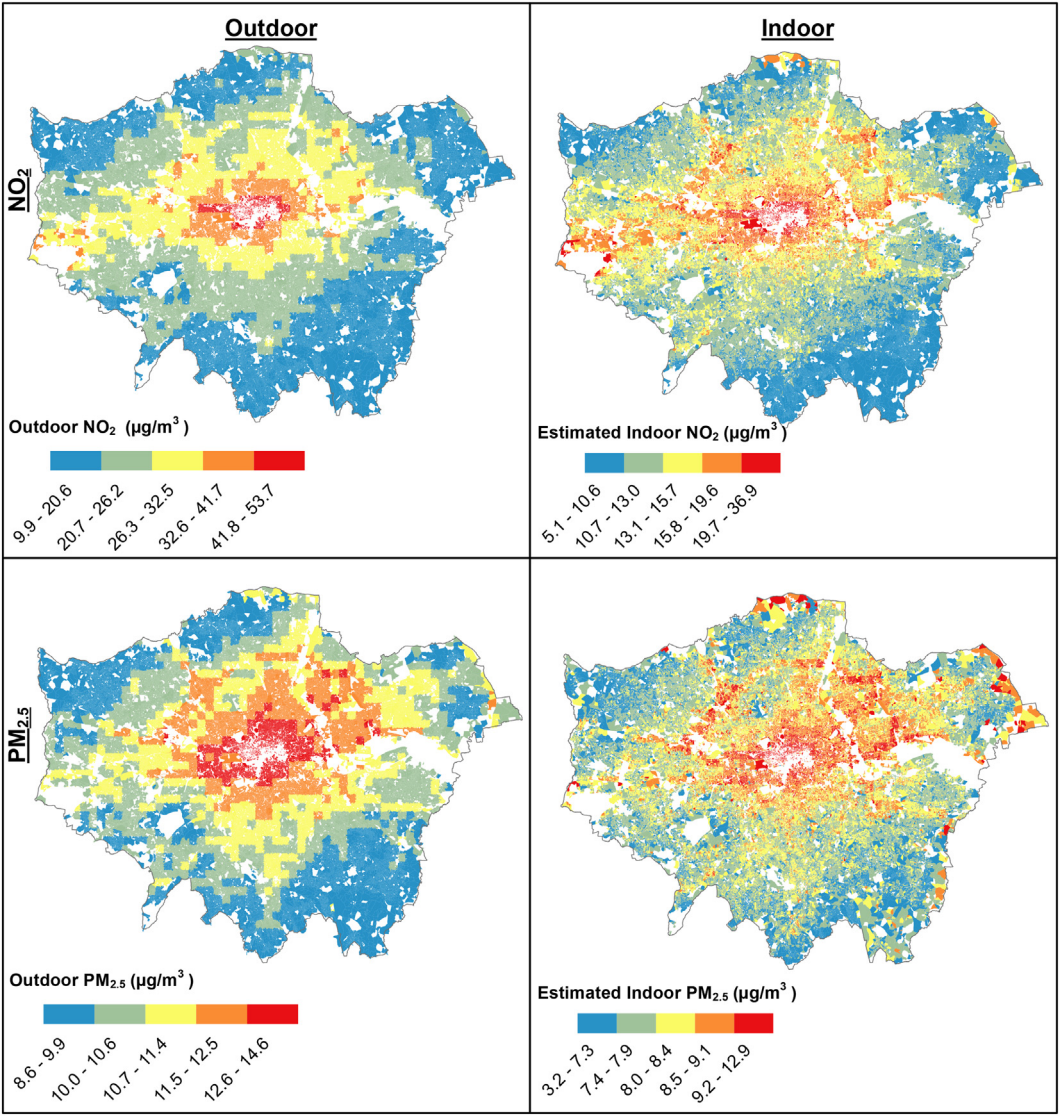
Postcode-average outdoor concentrations (left) and average estimated indoor concentrations (right) for PM_2.5_ and NO_2_ in London.

The modification of outdoor pollution exposure by housing for NO_2_ and PM_2.5_ can be seen in Fig. 5. For NO_2_, outdoor concentrations remain highest in central London; along the main train lines heading west of the city; and surrounding the North Circular road. The estimated indoor concentrations have a good spatial correlation with outdoor concentrations (ρ = 0.88), indicating that outdoor concentrations of NO_2_ may provide a reasonable estimate of relative indoor exposures to NO_2_ from outdoor sources. Indoor concentrations were estimated to be higher in Northern London due to high background levels of NO_2_, the prevalence of leaky detached or semi-detached dwellings with higher estimated air exchange rates, and in some cases greater exposure to wind due to the surrounding terrain.

For PM_2.5,_ the modifying effect of housing on exposure is stronger, likely due to the penetration factor, resulting in a significant change in exposure pattern relative to outdoor concentrations and less discernible spatial trends (ρ = 0.81). This indicates that outdoor PM_2.5_ levels may be a less reliable indicator of indoor exposure. Similar to NO_2_, elevated indoor concentrations were found in parts of Northern London due to high background levels, the housing stock, and the terrain.

### 3.2 National indoor CO concentrations

**Fig. 6:**
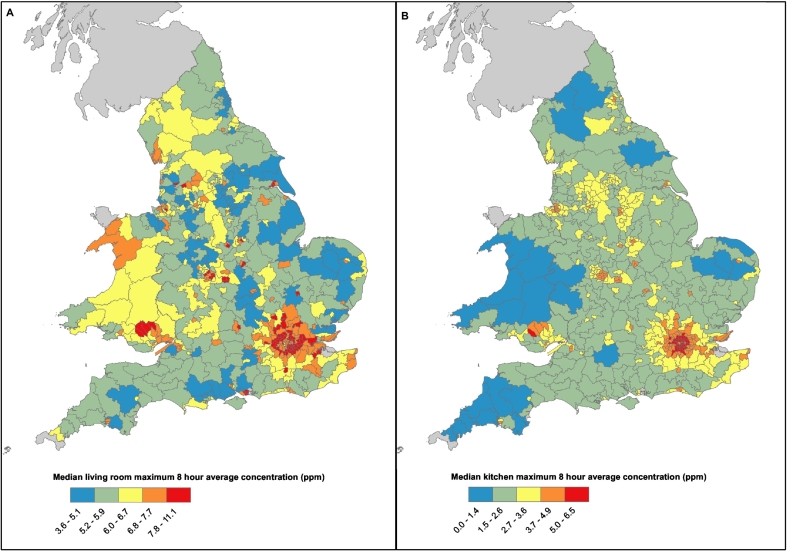
The constituency median annual maximum of the 8-hour rolling mean CO concentration (ppm) from indoor and outdoor sources in A) Living rooms with variable emissions from heating systems and weighted for smoking prevalence, and B) Kitchens, flagged by gas mains gas connectivity.

The metamodel was able to estimate indoor concentrations for all unique dwellings in the EPC database in around 5 h for each run (Laptop with Intel i5, 1.70 Ghz, 16GB RAM). Outdoor 8-hour average maximum concentrations for dwellings ranged from 0.2–1.8 ppm (99% CIs; median = 0.7 ppm). The equivalent current concentrations in individual buildings were much higher due to indoor sources, with concentrations in kitchens ranging from 0.4–9.9 ppm (99% CIs; median = 3.0 ppm) and living rooms, 0.3–25.6 ppm (99% CIs; median = 6.4 ppm). Estimated indoor concentrations of this demonstrative model can be seen in Fig. 6, with estimated areas of high exposure in urban areas such as London due to high outdoor background levels, and the metamodel predicting elevated levels of indoor-sourced CO due to the prevalence of flats and more modern air-tight housing. An estimated 38% of living rooms and 4% of kitchens had a maximum 8-hour mean concentration that exceeded the WHO guidelines for exposure during the course of a year under current conditions.

Energy efficiency changes to the underlying housing stock without adding additional ventilation is projected to lead to an increase in median CO concentration. Annual maximum 8-hour concentration under the retrofit scenarios for kitchens ranged from 0.5–8.6 ppm (99% CIs; median 3.6 ppm), while for living rooms it ranged from 0.3–26.3 ppm (99% CIs; median 7.2 ppm). This corresponds to a post-retrofit increase of 0.6 ppm and 0.8 ppm in kitchens and living roof, respectively. Following retrofit, an estimated 45% of living rooms and 6% of kitchens exceed the recommended WHO exposure thresholds.

## 4 Discussion

This paper described the development of a national housing stock model, and the application of an indoor air quality metamodeling framework in order to estimate the spatial variation in indoor air pollution. The combined housing stock model and metamodeling framework enables the rapid estimate of air pollution levels at the individual-dwelling address level for around half the English and Welsh housing stock. The tool may be used to estimate concentrations or exposures under current conditions, and following a range of adaptation scenarios, including changes to outdoor air pollution levels, reduced indoor emissions from changes in fuel use, as well as a variety of housing retrofit or new construction scenarios.

### 4.1 Housing stock

The EPC housing stock model offers a number of advantages over other UK publicly-available housing stock models. The 11.5 million dwellings in the parameterised dataset offers a significant improvement in coverage in comparison to the approximately 1 million dwellings from HEED that formed the basis of the national modelling work described by Taylor et al. (2016). For London, the 1.6 million dwellings in the EPC represent an improvement in both coverage and building information provided by the underlying building stock data in Taylor et al. (2014). The spatial information held in the database allows the geographical modification of pollution exposure to be considered, providing an advantage over datasets such as the EHS which have limited spatial information. The EPC dataset is, however, limited by the lack of occupant information such as the presence of smokers, which is available in some versions of the EHS.

The comparison between the EPC dataset and the EHS (Appendix 2) showed that the EPC data is reasonably representative of the English housing stock. However, there are a number of potential sources of bias or error in the EPC dataset, including:

• EPC certificates are obtained when buildings are constructed, retrofit, sold, or rented. This may mean a bias towards more energy efficient dwellings, while housing that is not retrofit and has not been included in a transaction will be underrepresented.
• There are a number of concerns regarding the quality of EPC surveys. Where obvious errors were found in the data, the data was removed. However, due to the large number of dwellings in the EPC dataset, it was not possible to check the estimated building parameters for each dwelling.

Nonetheless, the EPC dataset has valuable potential as a source of dwelling information, and as a platform for modelling the dwelling modification of environmental hazard exposures.

### 4.2 PM_2.5_ and NO_2_

The metamodeling framework enabled the rapid calculation of I/O ratio and indoor concentrations of pollution from indoor sources. The I/O ratios of PM_2.5_ modelled here are broader in range (0.13–0.86) than the mean I/O ratios factors found in previous modelling work for London (0.45–0.62), and empirical studies in Europe (0.30–0.70) (Hänninen et al., 2011) and internationally (0.3–0.82) (Chen and Zhao, 2011), although the range from 1st to 99th percentile is similar (0.53–0.73). This is likely due to the very wide range of housing variants modelled in this study. Similarly, the range of postcode-average indoor PM_2.5_ from outdoor sources estimated here (3.2–12.9 μg m^−3^) is much broader than the 5.2–11.4 μg m^−3^ estimated in Taylor et al. (2014), while the medians were similar (8.0 μg m^−3^ versus 7.9 μg m^−3,^ respectively). This is due to the larger range in building variants modelled, better coverage of housing data, and the smaller spatial unit of aggregation possible due to this improved coverage. Results indicate that the maximum I/O ratio for PM_2.5_ is six times greater than the minimum, while the maximum I/O ratio for NO_2_ is seven times greater than the minimum, demonstrating the significant potential modification of outdoor pollution exposure from housing. Outdoor NO_2_ may be used as a reasonable proxy for indoor exposures to outdoor levels, while outdoor PM_2.5_ has a lower correlation with the corresponding indoor levels.

The tool is flexible and may be run in the future with any pollutant, given a deposition velocity, and penetration factor. We acknowledge there is a great deal of uncertainty and limitations in the modelling of these pollutants. The I/O model calculates an annual average, however it is likely that the I/O ratio changes seasonally due to climate, for by example increased window-opening during summer, and greater wind pressures during winter. Occupant behaviour is modelled deterministically, including window-opening that occurs over a static indoor temperature threshold. This does not allow for variation in window-opening behaviour by occupants – for example due to personal preference, or a reluctance to open due to proximity to busy roads or areas of high crime. The model would, however, be capable of doing so should more established evidence on temperature and location related window-opening behaviour become available. Additionally, the occupant schedule within dwelling microenvironments is fixed, with exposures estimates based on the presumed location of an occupant within the dwelling. We assume that occupants are home during the day, reflecting the housing modification of exposure rather than absolute occupant exposure. While housing is an important microenvironment for exposure (Smith et al., 2016), occupants may not be home during peak hours of outdoor air pollution levels. There is also significant uncertainty in pollution deposition, penetration factors, and the modelled housing characteristics. Further evaluation of the model sensitivity to variations in inputs should be performed using a global sensitivity analysis (Das et al., 2014).

There were also a number of limitations with the outdoor air pollution data. We do not include temporal variation in outdoor levels, and the relatively coarse grid of background levels may not reflect actual outdoor levels close to major roads, for example. We have also assumed that outdoor air pollution does not vary with the height of the building, and that top floor flats will be exposed to the same level of outdoor air pollution as ground floor flats, whereas stratification of pollution may occur by busy roads. As the I/O ratio is converted to a total indoor concentration during a post-processing step, it would be possible to include this in the future. There remains a lack of empirical data at the required scale with which to validate the model outcomes.

While the modelled results have been summarised at postcode-level, it is not known what spatial resolution is required to estimate population exposure reliably. Aggregating at larger spatial units may have the effect of reducing errors caused by model assumptions and unreliable building input data, as well as helping to minimise the temporal limitations of the model (Stroh et al., 2007). However, outdoor air pollution and housing in London may vary significantly over small geographical distances, which is likely to have significant implications for exposure.

### 4.3 CO

The model of indoor CO is intended to illustrate the application of the model to a spatially-varying housing stock, where housing characteristics may influence exposure risks. Uncertainty is particularly large for indoor air pollution from indoor sources. A range of values for emission rates and deposition velocities may be found in the literature for different pollutants, which will lead to a range in indoor concentration estimates. We have not quantified this uncertainty here, but the ability to vary the emissions and deposition velocity means that this can be carried out in future studies.

The potential for the model to account for occupant behaviour on indoor concentration is limited: while it can modify the temperatures above which windows may be opened, the use of extract fans, and can turn emission sources on or off, emission schedules, locations, and sources are modelled deterministically. This means that for CO concentrations, the emission from heaters is currently fixed to the living room, while some important potential sources of CO – such as from attached garages – are not modelled. The model is highly sensitive to housing characteristics and pollutant emission assumptions, which should be explored in further studies using stochastic methods. The model could be further improved by using distributions of occupancy behaviours based on empirical data, for example thermostat settings (Shipworth et al., 2010). We assume an extract ventilation of 60 l/s in the kitchen during cooking, in-line with building regulations (HM Government, 2010) for extracts that are not adjacent to the hob. This likely means an under-estimate of CO levels in the 51% of English dwellings that do not have a working extract fan (DCLG, 2011), and an over-estimate in dwellings that have an extract fan adjacent to the hob. Future versions of the model could test different extract fan ventilation rates and locations within the kitchen.

When applying the model to a building stock, it is potentially misleading to predict occupant pollutant-generating behaviours to the building-level. We assumed ‘average’ behaviours, assuming that deviations from this average would be reduced when the results were aggregated across spatial units. Estimates of pollution from indoor sources at the individual-building level should therefore be treated with caution, and viewed as an illustrative estimate of the potential housing modification of exposure rather than absolute estimates of concentration. As with I/O ratios, the model is largely theoretical, and there is a limited amount of empirical data on which to validate the results.

The metamodel has been applied here to estimate indoor air pollution, but is also capable of modelling annual space heating energy use, indoor overheating, standardised indoor temperature (SIT), and moisture. The EPC database provides important housing data which may also help inform other studies, such as being used to identify emission sources due to fuel burning, locations for potential energy efficiency interventions, and linking housing data to health records. By applying the metamodeling framework to the parameterised EPC dataset, we have produced spatially-varying indoor pollution estimates that may be used in exposure assessments and epidemiological studies. The ability to rapidly run the metamodel for the national housing stock means that the indoor air pollution implications of housing policies may be evaluated, while also potentially accounting for modelled future changes in outdoor exposure. Future research will include expanding the model's capacity to simulate pollutants from indoor sources, and applying the exposure estimates in health models under current and future scenarios. Further development of the model's ability to account for variations in occupant schedules – for example through Markov Chain models – would enable the role of occupant behaviour to be accounted for, thereby enabling the range of population indoor exposures to be better understood.

## 5 Conclusions

We have described the application of a metamodelling framework to predict indoor concentrations of PM_2.5_ and NO_2_ from outdoor sources, and indoor concentrations of CO from both indoor and outdoor sources. The EPC building stock data improves the spatial coverage and buildings information relative to housing models used previously as the basis for modelling studies, while the metamodeling approach makes it computationally possible to estimate indoor air pollution concentrations for individual-dwellings at a national scale. We predict median I/O ratios for London dwellings of 0.41 (99% CIs; 0.34–0.60) for NO_2_, and 0.60 (99% CIs; 0.53–0.73) for PM_2.5_. These result in estimated median indoor exposures to outdoor-sourced NO_2_ of 12.9 μg m^−3^ (99% CIs; 7.3–23.0 μg m^−3^), and for PM_2.5_ of 8.0 μg m^−3^ (99% CIs; 6.4–10.2 μg m^−3^). Housing is shown to have an important modifying effect on exposure to outdoor pollutants, with the effect stronger for PM_2.5_ (ρ = 0.81) than for NO_2_ (ρ = 0.88).

While highly sensitive to model input assumptions, the demonstrative CO model estimated indoor concentrations of CO from both indoor and outdoor sources to have a national median of 3.0 ppm (99% CIs; 0.4–9.9 ppm) in kitchens and 6.4 ppm (99% CIs; 0.3–25.6 ppm) in living rooms; complete retrofit without additional purpose-provided ventilation was estimated to increase exposure in both rooms 0.6 ppm and 0.8 ppm, respectively. Indoor exposures to CO were predicted to be greatest in urban areas, due to the prevalence of flats with lower air change rates trapping indoor generated air pollution, as well as high outdoor concentrations. Modelling building modification of pollutant exposure over a spatially distributed building stock enables the estimate of exposures for a population spending significant amounts of time indoors, and can enable locations of potentially elevated exposures to be identified.

## Capsule

We use a model derived from building physics simulations to estimate 1) the indoor concentration of NO_2_ and PM_2.5_ from outdoor sources for 1.6 million dwellings in London, and 2) CO from both indoor and outdoor sources for 11.5 million dwellings across England and Wales at individual-building level; results are then mapped to show the spatial variation in indoor concentration.

## Author statement

Jonathon Taylor: Conceptualization, Methodology, Investigation, Formal Analysis, Data Curation, Writing – Original Draft, Visualization. Clive Shrubsole: Conceptualization, Methodology, Writing – Review & Editing, Funding Acquisition. Phil Symonds: Conceptualization, Software, Methodology, Writing – Review & Editing. Ian Mackenzie: Formal Analysis, Data Curation, Writing – Review & Editing. Mike Davies: Resources, Writing – Review & Editing, Supervision, Project Administration, Funding Acquisition.

## Supplementary data

Supplementary material Image 1

## Footnotes

2 Particulates with a diameter < 2.5 μm.
