# Supplementary material for: Application of an indoor air pollution metamodel to a spatially-distributed housing stock
Source: Sci Total Environ. 2019 Jun 1;667:390–9. doi: 10.1016/j.scitotenv.2019.02.341 (PMC6467545; doi:10.1016/j.scitotenv.2019.02.341)
Supplement: Supplementary file 1 — Supplementary material [file mmc1.docx]

**Appendix 1 – Dwelling Archetypes**

**
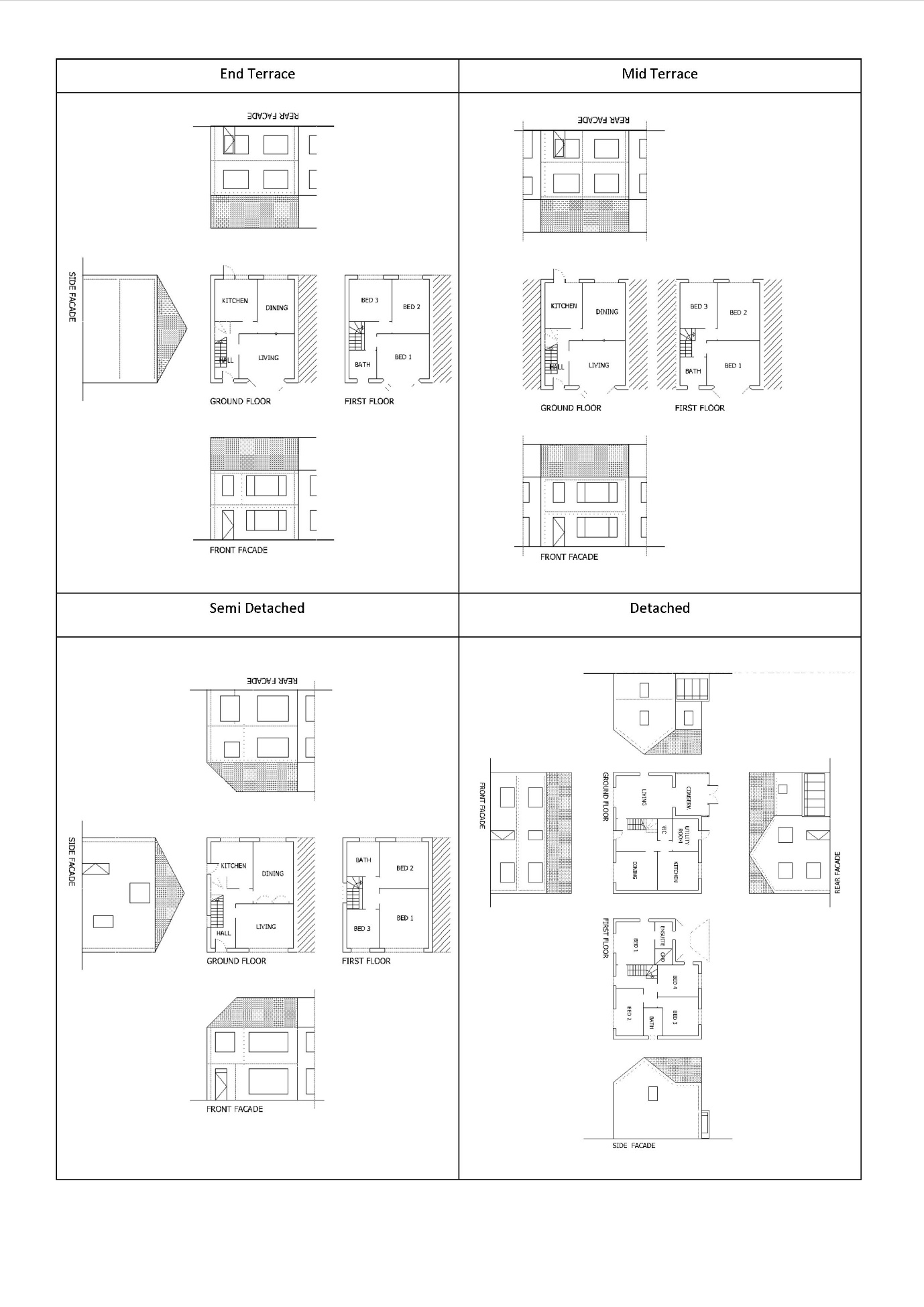
**

Figure A1.1. Housing archetypes (page 1)

**
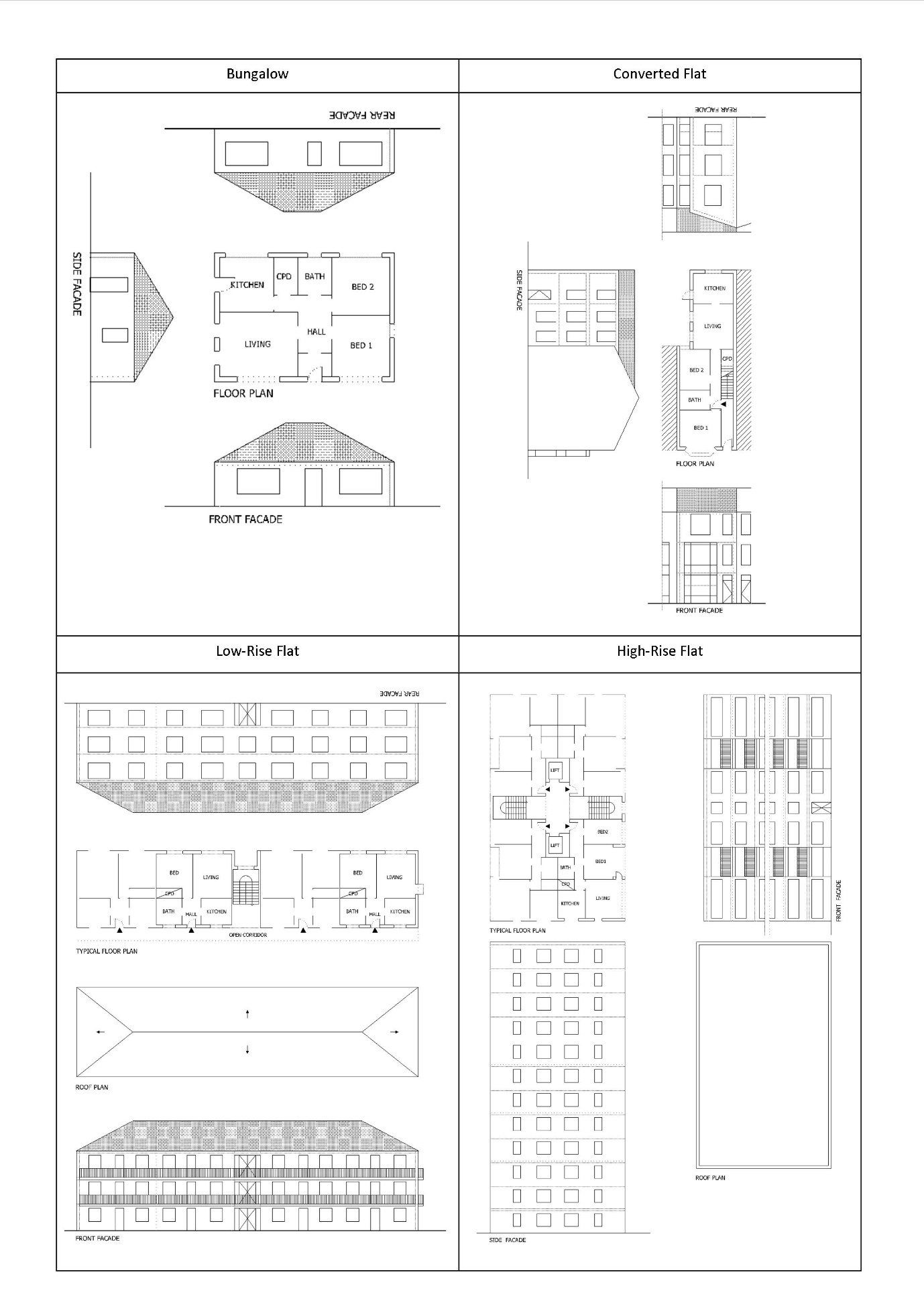
**

Figure A1.2. Housing archetypes (part 2)

**Appendix 2 - EPC Parameterisation**

The metamodeling framework requires as input information on building characteristics (Table A2.1). The metamodel, generated from a large number of EnergyPlus simulations, then calculates a number of indoor temperature, air pollution, moisture, and space-heating energy use based on these parameters. The conversion of EPC data required a number of assumptions, which are described as follows. Where possible with the data held with EPC, conversion was done in line with the guidance provided in the DECC document “*Converting English Housing Survey Data for Use in Energy Model*s” [1]. This conversion methodology has been used previously on the 2010-2011 EHS; there is therefore another dataset of English dwellings with which to compare the results. All conversion and dataset analysis was done in SAS 9.4. The ranges for emission rates were taken from the PANDORA [2] database for multiple indoor pollutants. The distribution of these emission rates was multimodal so we ended up assuming a uniform distribution, which is preferential where there is a high degree of uncertainty.

| **Parameter** | **Range** | **Distribution** |
| --- | --- | --- |
| Wall U-value | 0.15-2.55 W/m^2^K | Uniform |
| Roof U-value | 0.10-2.25 W/m^2^K | Uniform |
| Window U-value | 0.85-4.80 W/m^2^K | Uniform |
| Floor U-value | 0.15-1.30 W/m^2^K | Uniform |
| Fabric air permeability | 0-∞ m^3^/h/m^2^ @ 50 Pa | Truncated Normal (*µ*=20*,*σ=10) |
| Orientation | 0-360^◦^ | Uniform |
| Terrain Type | City/Urban/Rural | Discrete |
| Floor area scale factor | 0.65-2 | Uniform |
| Floor height | 2-3*m* | Uniform |
| Glazing faction | 0.1-0.6 | Uniform |
| Occupant window opening temperature threshold | 10-∞^◦^C | Truncated Normal (*µ*=24*,*σ=5) |
| Occupant thermostat setting | 15-26 ^◦^C | Truncated Normal (*µ*=22*,*σ=3) |
| Internal gains scale factor | 0.35-1.9 | Uniform |
| CO emission rate (kitchen) | 0.05-0.70 ×10^−6^*m*^3^*/s* | Uniform |
| Extract fan efficiency (kitchen) | 0-1 | Uniform |
| CO emission rate (living room) | 0.05-1.0 ×10^−6^*m*^3^*/s* | Power law |

**Table A2.1.** Model input parameters sampled within the LHE with range and distributions used.

- - 1. Dwelling characteristics

Dwelling types were classified based on the EPC parameters *Property Type*, *Built Form*, *Flat Storey Count*, *Floor Level*, and *Flat Top Storey* based on the following assumptions:

1. Where the *Property Type* is ‘House’, the *Built Form* describes the house type as end terrace, mid terrace, semi-detached, detached, or bungalow. Enclosed terraces were treated as unenclosed terraces.
2. Where *Property Type* is ‘Flat’ or ‘Maisonette’, and the *Built Form* refers to a type of house, above, then it was assumed to be a converted flat.
3. Where *Property Type* is ‘Flat’ or ‘Maisonette’ in an unconverted house and *Flat Storey Count* is 3 or less, then it was assumed to be a low-rise purpose-built flat. Where *Flat Storey Count* is greater than three, it was assumed to be a high rise purpose-built flat.
4. For flats and maisonettes, *Floor Level* was used to classify dwellings as Bottom, Middle, or Top floor flats.
   - 1. Fabric U-Value

The metamodel requires U-values for walls, floors, roofs, and windows. U-values were assigned using the methods described in [1] and SAP 2009 [3] as a reference.

***Windows***

The *Windows Energy Efficiency* variable classifies windows as ‘Very Good’, ‘Good’, ‘Average’, ‘Poor’, or ‘Very Poor’. These were converted into U-values based on conversion information provided by BRE [4]. The metamodel automatically assumes that all windows with U-values that correspond to post-2002 double glazing and triple glazed windows have trickle vents.

***Roofs***

For roofs, the U-values were determined based on the following method:

1. Roof types were classified based on text strings in *the Roof Description* column (pitched, flat, or thatched).
2. When the *Roof Description* column contained a number followed by ‘mm’, it is assumed to be a thickness of insulation, and the number is extracted. When the *Roof Description* column contains the string ‘no insulation’, then insulation thickness is assumed to be zero.
3. When the *Roof Description* column contains the term ‘thermal transmittance’, the number was extracted.
4. When there was a thermal transmittance value, this was used as the U-value for the roof. Otherwise, the U-value was assumed from the type of roof and insulation thickness from SAP Table S10. Finally, these U-values were compared against the *Roof Energy Efficiency* variable in the EPC; if they fell within the U-value ranges specified by [4] for ‘Very Good’, ‘Good’, ‘Average’, ‘Poor’, or ‘Very Poor’ roofs, then the values were kept; otherwise they were assumed to be erroneous, and the dwellings were assigned U-values based on the *Roof Energy Efficiency* classification.

***Walls***

For walls, the classification methodology was as follows:

1. The column *Walls Description* was searched for keywords (cavity, system, solid brick, cob, timber, sandstone, or granite) to classify the walls.
2. The *Walls Description* column was also searched for keywords including ‘no insulation’, ‘insulated’, ‘filled’, ‘external insulation’, ‘internal insulation’, and ‘partial insulation’.
3. Where the *Walls Description* column contained the term ‘thermal transmittance’, this number was extracted as the U-value.
4. Where there is no thermal transmittance provided, the wall type and insulation were used to determine the row in SAP Table S6 that applied. The *Wall Energy Eff* column was then used to estimate a U value for the wall based on the text description in the column (very poor, poor, average, good, very good) and the range of values in SAP table S6 column for each wall type and insulation level. Finally, these U-values were compared against the *Wall Energy Efficiency* variable in the EPC; if they fell within the U-value ranges specified by [4] for ‘Very Good’, ‘Good’, ‘Average’, ‘Poor’, or ‘Very Poor’ walls, then the values were kept; otherwise they were assumed to be erroneous, and the dwellings assigned U-values based on the *Wall Energy Efficiency* classification.

A similar process was followed to determine the thickness of the walls using SAP Table S3.

***Floors***

For floors, the following process was used to determine their U-value:

1. Floors were classified as solid or suspended based on whether the *Floor Description* column contains those key words.
2. The presence of insulation in the floors was determined using the presence of the keywords ‘no insulation’, ‘insulated’, or ‘limited insulation’ in the *Floor Description* column.
3. Where the *Floor Description* column contains the term ‘thermal transmittance’, this number was extracted as the U-value.
4. Where the *Floor Description* column contains a number followed by ‘mm’, this was taken to be a thickness of insulation.
5. Floor U-value was then calculated using the methodology described in SAP Section S5.4 using the above-estimated thickness and U-values of the walls, the floor type, insulation level, and an estimate of the area of the building (determined from the estimated number of floors in the dwelling and data from the *Total Floor Area* column) and periphery of the building (estimated from the *Total Floor Area*, assuming dwellings are square). Finally, these U-values were compared against the *Floor Energy Efficiency* variable in the EPC; if they fell within the U-value ranges specified by [4] for ‘Very Good’, ‘Good’, ‘Average’, ‘Poor’, or ‘Very Poor’ floors, then the values were kept; otherwise they are assumed to be erroneous, and the dwellings are assigned U-values based on the *Floor Energy Efficiency* classification.
   - 1. Glazing fraction

The EPC contains very approximate information on the amount of glazing within each dwelling, classified as ‘very low’, ‘low’, ‘average’, ‘high’, and ‘very high’. The EHS was used to convert these values into glazing ratios. For each region and built form type, the average, 10^th^, 30^th^, 70^th^, and 90^th^ percentile glazing ratio was calculated from EHS data and used to represent the ‘average’, ‘very low’, ‘low’, ‘high’, and ‘very high’ classifications, respectively. These glazing fractions were then joined to the EPC dataset.

- - 1. Permeability

Permeability was calculated following SAP Ventilation Rate calculations [1,2]. The following methodology was used:

1. Infiltration from flues, fans, chimneys, and vents: Chimney infiltration was estimated from *Number of Open Fireplaces*. The infiltration from vents was calculated using the *Mainheat Description* and *Secondheat Description*, where those containing the terms ‘open flue’, ‘oil’, ‘solid’, ‘wood’, ‘coal’, ‘anthracite’, or ‘gas’ were assumed to have an open flue. The number of intermittent fans was estimated using SAP table S5, which related the number of fans to the number of habitable rooms and dwelling age band. Here, we use the EPC column *Number Habitable Rooms* and, as a proxy for age, estimated wall U-value. Infiltration from passive vents was ignored, but instead was modelled in the physics tool as trickle vents dependent on the window type. Dwelling volume was taken to be the *Total Floor Area* times the *Floor Height*.
2. Additional Infiltration: This assumed houses and maisonettes were two storeys and flats one storey.
3. Structural infiltration was estimated based on whether the *Walls Description* column contains ‘timber’, ‘system’, or neither.
4. Floor infiltration: Estimated based on the floor type (solid or suspended), described above.
5. Draught Lobby infiltration: Flats and maisonettes were assumed to have a draught lobby, while all other dwellings were not.
6. Window infiltration: Here, it was assumed that all double, secondary, and triple glazed windows had draught proofing. The extent of the draught proofing was modified based on key terms in the *Windows Description* column including ‘some’, ‘partial’, and ‘mostly’, which was assumed to mean 33%, 50%, and 66%, respectively. Without those key terms, it was assumed that 100% of windows with double, secondary, or triple glazing had draught proofing.
7. The various infiltration sources were summed as per SAP. The bounding surface area of the dwelling was estimated based on the assumption that dwellings had a square footprint; the surface area to volume ratio was then used to convert infiltration to a permeability (m^3^/h/m^2^), based on the ‘rule of 20’ assumption that converts between infiltration and a pressurised air change rate.
   - 1. Terrain type

The metamodel requires input of the wind exposure of the building based on the surrounding terrain. As per the modelling work described in Taylor et al [5], the Office for National Statistics (ONS) urban-rural classifications were used to define the surrounding terrain based on the building location [6]. The postcode of each EPC dwelling was used to locate dwellings within a 2011 Census Output Area (COA) based on the centroid of the postcode. The ONS urban/rural classification was then used to classify each dwelling according to their surrounding area (city, urban, or rural).

1. Evaluation of parameterised EPC data
   1. Spatial variation

The spatial variation in the modal EPC dwelling characteristics can be seen in Figures A2.1 and A2.2, showing pockets of poorly-insulated dwellings in London in particular. Urban areas are also dominated by dwellings with smaller floor areas relative to rural areas.


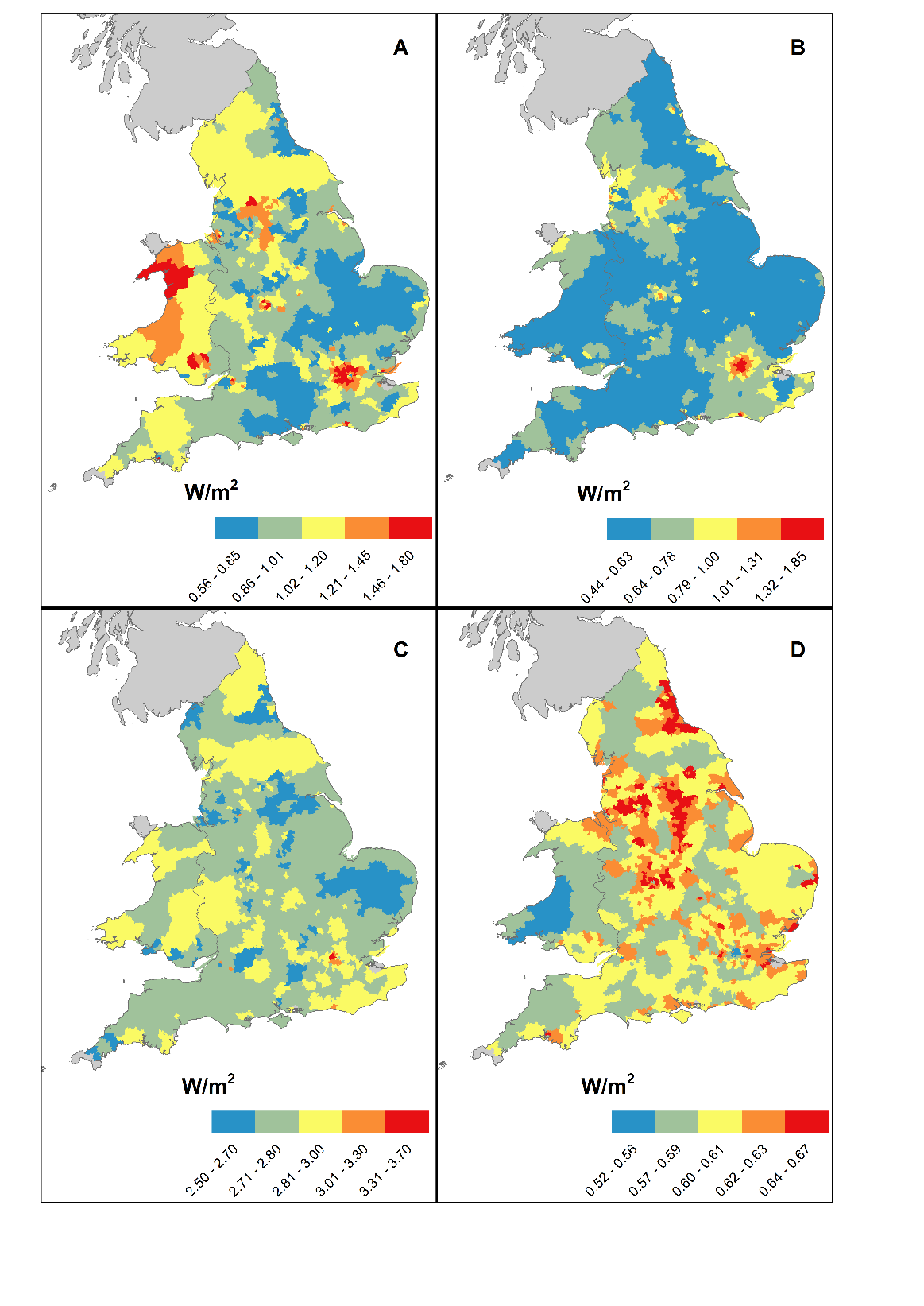


Figure A2.1. Estimated modal U-values of building fabrics, including A) External walls, B) Roofs, C) Windows, and D) Floors.


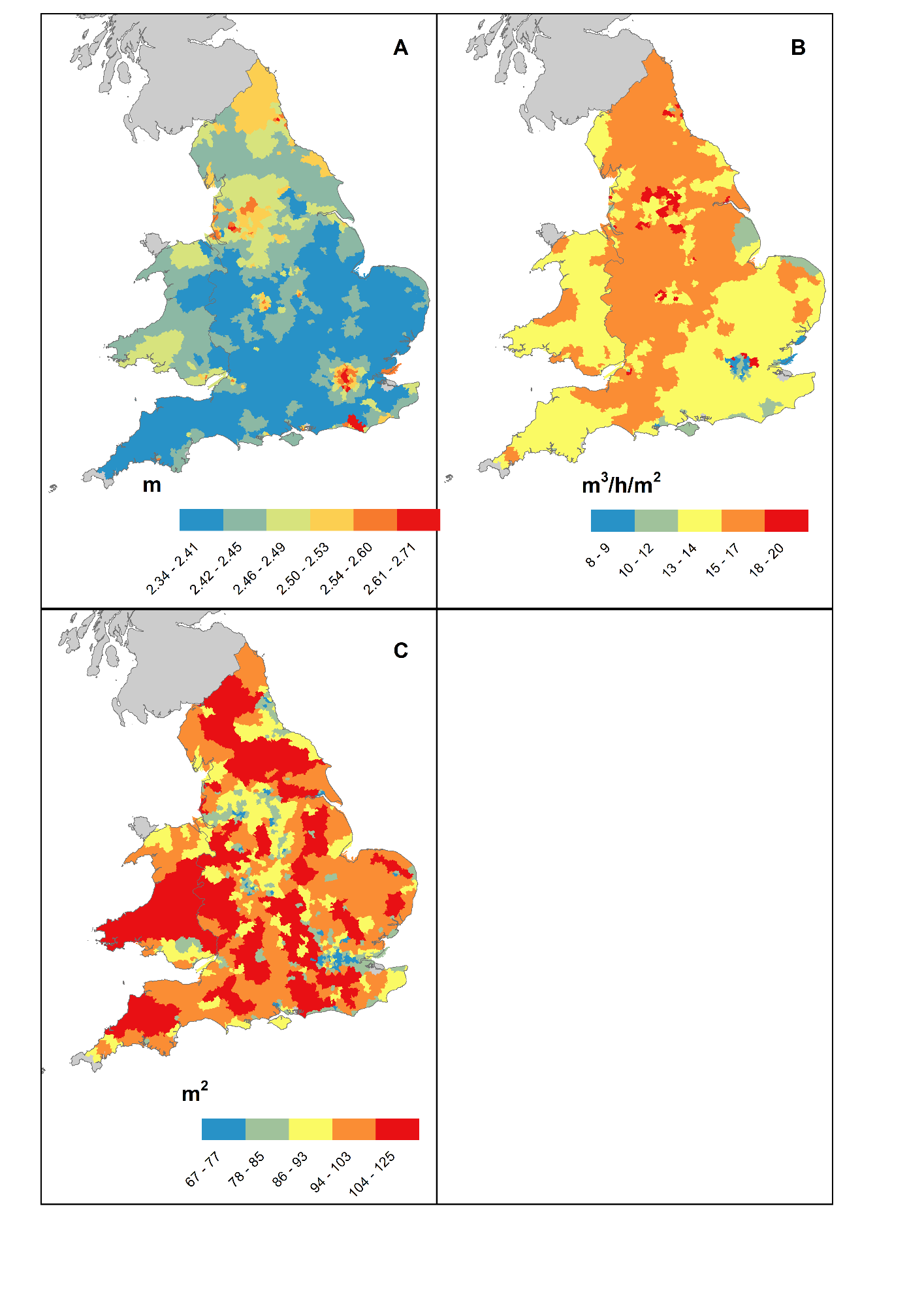


Figure A2.2. Mean estimates of A) Ceiling height, b) Permeability, and C) Floor Area by constituency.

- 1. Comparison to EHS data

A random stratified (by English Government Office Region, GOR) sample of parameterised EPC data was compared to the EHS dataset, a representative sample of dwellings in England (Figures A2.3 to A2.7). Generally, the EPC data shows similar distributions to the parameterised EHS data. Differences include the following:

- For U-values, distributions were broader due to the specific u-values provided in the EPC dataset, while the EHS data had peaks at certain values due to the use of SAP lookup tables. Peaks occurred in roughly the same location, although there was a slight skew towards more energy efficient building fabrics in the EPC datasets.
- The permeability of the EPC dwellings also showed similar distributions, albeit with a slight skew towards dwellings with reduced permeability relative to the EHS dataset.

The results provide confidence that the parameterised EPC dataset is representative of the English housing stock, albeit with minor biases towards energy efficient dwellings.


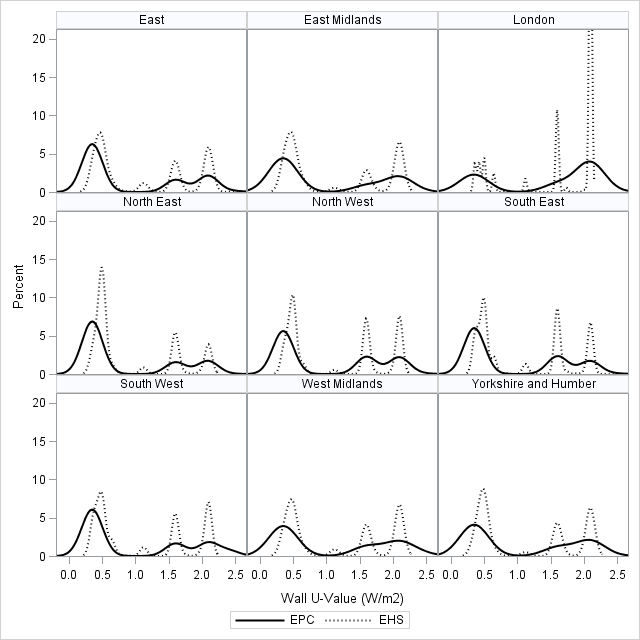


Figure A2.3. A comparison of a random selection of EPC dwellings (1,000,000) to the representative EHS dwellings, by English region, for wall U-value.


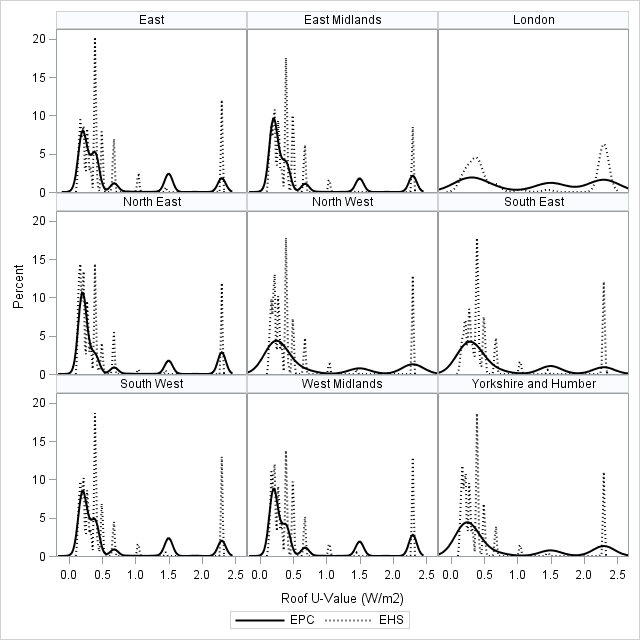


Figure A2.4. A comparison of a random selection of EPC dwellings (1.000,000) to the representative EHS dwellings, by English region, for roof U-value.


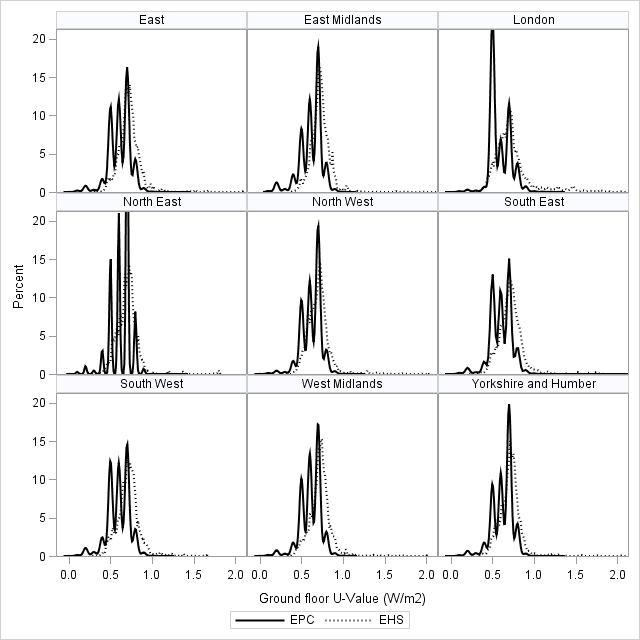


Figure A2.5. A comparison of a random selection of EPC dwellings (1,000,000) to the representative EHS dwellings, by English region, for ground U-value.


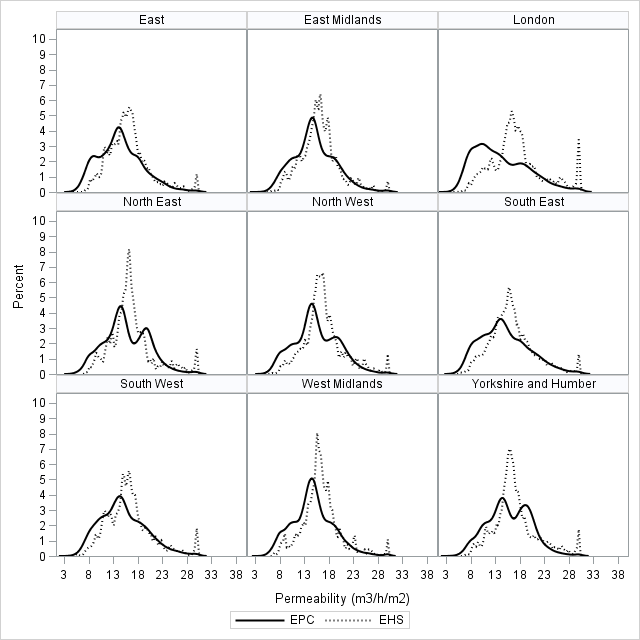


Figure A2.6. A comparison of a random selection of EPC dwellings (1,000,000) to the representative EHS dwellings, by English region, for dwelling permeability.


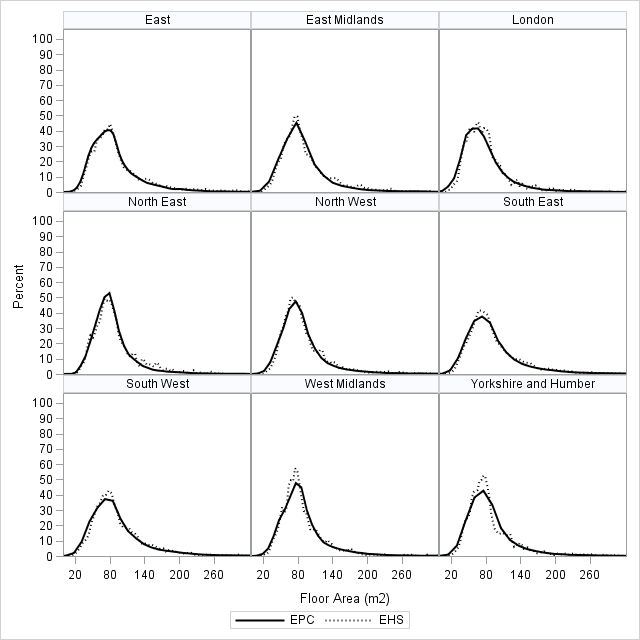


Figure A2.7. A comparison of a random selection of EPC dwellings (1,000,000) to the representative EHS dwellings, by English region, for dwelling floor areas.

References

1. M. Hughes, P. Armitage, J. Palmer, A. Stone, Converting English Housing Survey Data for Use in Energy Models, Department of Energy and Climate Change, London, UK, 2012.
2. M.O. Abadie, P. Blondeau, PANDORA database: A compilation of indoor air pollutant emissions, HVAC&R Res. 17 (2011) 602–613.
3. BRE, The Government’s Standard Assessment Procedure for Energy Rating of Dwellings, Building Research Establishment, Watford, UK, 2009.
4. Henderson, J., Personal Communication of BRE report “*Descriptions and energy banding, SAP and RdSAP 9.92”, Building Research Establishment, Watford, 14/07/2007.*
5. J. Taylor, M. Davies, A. Mavrogianni, C. Shrubsole, I. Hamilton, P. Das, B. Jones, E. Oikonomou, P. Biddulph, Mapping indoor overheating and air pollution risk modification across Great Britain: A modelling study, Build. Environ. (2016). doi:10.1016/j.buildenv.2016.01.010.
6. Office for National Statistics, 2011 rural/urban Classification for Small-area Geographies, Office for National Statistics, London, 2011.
